# Complete genome analysis of a *Siphoviridae* phage TSK1 showing biofilm removal potential against *Klebsiella pneumoniae*

**DOI:** 10.1038/s41598-018-36229-y

**Published:** 2018-12-17

**Authors:** Rabia Tabassum, Muafia Shafique, Komal Amer Khawaja, Iqbal Ahmed Alvi, Yasir Rehman, Cody S. Sheik, Zaigham Abbas, Shafiq ur Rehman

**Affiliations:** 1 Department of Microbiology and Molecular Genetics, University of the Punjab, Lahore, Pakistan; 2 Swenson College of Science and Engineering, University of Minnesota Duluth, Duluth, USA

## Abstract

Multidrug-resistant *Klebsiella pneumoniae* is a nosocomial pathogen, produces septicemia, pneumonia and UTI. Excessive use of antibiotics contributes towards emergence of multidrug-resistance. Bacteriophage-therapy is a potential substitute of antibiotics with many advantages. In this investigation, microbiological and genome characterization of TSK1 bacteriophage and its biofilm elimination capability are presented. TSK1 showed narrow host range and highest stability at pH 7 and 37 °C. TSK1 reduced the growth of *K*. *pneumoniae* during the initial 14 hours of infection. Post-treatment with TSK1 against different age *K*. *pneumoniae* biofilms reduced 85–100% biomass. Pre-treatment of TSK1 bacteriophage against the biofilm of *Klebsiella pneumoniae* reduced > 99% biomass in initial 24 hr of incubation. The genome of TSK1 phage comprised 49,836 base pairs with GC composition of 50.44%. Total seventy-five open reading frames (ORFs) were predicted, 25 showed homology with known functional proteins, while 50 were called hypothetical, as no homologs with proved function exists in the genome databases. Blast and phylogenetic analysis put it in the *Kp36 virus* genus of family *Siphoviridae*. Proposed packaging strategy of TSK1 bacteriophage genome is headful packaging using the pac sites. The potential of TSK1 bacteriophage could be used to reduce the bacterial load and biofilm in clinical and non-clinical settings.

## Introduction

*Klebsiella pneumoniae* is an important biofilm-forming bacteria responsible for a broad range of infections, placing it among the eight most significant nosocomial pathogens^1^. It is ubiquitous and causes septicemia, pneumonia and urinary tract infections. It is one of the most important nosocomial pathogen causing 5–7% hospital-acquired infections and resists a wide range of antibiotics^2^. *K*. *pneumoniae* have two main resistance mechanisms: production of enzymes and biofilm formation. Since 1980, the third-generation cephalosporins (cefotaxime, ceftazidime, and ceftriaxone) have been introduced as useful and effective drugs against most nosocomial infections. The excessive use of cephalosporins in clinical settings and production of a β-lactamase enzyme is the leading cause of antibiotic resistance in *K*. *pneumoniae*^3^.

Due to the emergence of cephalosporin-resistant *K*. *pneumoniae* strains, carbapenems were broadly used for the treatment of serious infections caused by multidrug-resistant *K*. *pneumoniae*^4^. However, in recent years, widespread use of carbapenems and fluoroquinolones have accelerated the global emergence of its resistant strains. Carbapenem-resistant *K*. *pneumoniae* (CRKP) is an emerging antibiotic-resistant nosocomial pathogen^5^. The acquisition of genes encoding enzymes capable of breaking down most β-lactams including carbapenems (*K*. *pneumoniae* carbapenemases (KPCs), carbapenemases of the oxacillinase-48 (OXA-48) and New Delhi Metallo-β-lactamase (NDM) carbapenemases) leads to develop antibiotic resistance against carbapenems^6^. Fluoroquinolones having broader antimicrobial spectra and improved pharmacokinetic properties are extensively used in the treatment of the most nosocomial infections. *K*. *pneumoniae* develops resistance against quinolones by incorporating mutations in enzymes (DNA gyrase and topoisomerase) which are targets of these antibiotics^7^. The extensive use of fluoroquinolones in treating bacteremia and UTI infections are responsible for decreased susceptibility of *K*. *pneumoniae* against them^8^.

Biofilm is the heterogeneous, organized microbial communities that are entrenched in a hydrated self- produced matrix^9^. It provides a shield from desiccation and allows bacteria to achieve 1000 times higher densities than the freely existing forms of bacteria. Due to the presence of capsular polysaccharide (CPS) and lipopolysaccharide (LPS), *K*. *pneumoniae* biofilm, safeguard it against the host immune system^10^. These diverse microenvironments confer properties of multidrug resistance, protection against the opsonization and other immune reactions^11^. The threat of antibiotic resistance and inability of breaking the biofilm structure necessitates to explore novel strategies for controlling biofilm producing pathogens^1^. Bacteriophage therapy is a viable option of controlling bacterial entities involved in biofilm formation^12^. It is preferable over antibiotic treatment, due to its high specificity and self-replication at the site of infection along with its narrow host-range that leaves normal flora unharmed^13^. Through their interaction with host cells, phage-borne exopolysaccharide depolymerases degrade biofilm matrix that acts as a barrier for antimicrobial agents, infects the bacterial cells and may cause extensive biofilm disruption^9^.

The global emergence of extended-spectrum beta-lactamases (ESBLs), carbapenem and fluoroquinolones resistance in *K*. *pneumoniae* is a serious problem and only a few remaining last-resort antibiotics can be used to treat fluoroquinolones and carbapenem-resistant *K*. *pneumoniae* (CRKP) infections^14,15^. The worldwide spread of *Klebsiella pneumoniae* spp., resistant to a variety of antibiotics, threatens to revert modern medicine to a pre-antibiotic era. This research was conducted to isolate a novel virulent bacteriophage against multidrug-resistant *K*. *pneumoniae*. The isolated bacteriophage (TSK1) was characterized based on morphology, physiological parameters, host range and genome characteristics. Furthermore, the tendency of bacteriophage to disintegrate the biofilm was also assessed.

## Results

### TSK1 bacteriophage forms opaque halo zone around clear plaques

**Figure 1:**
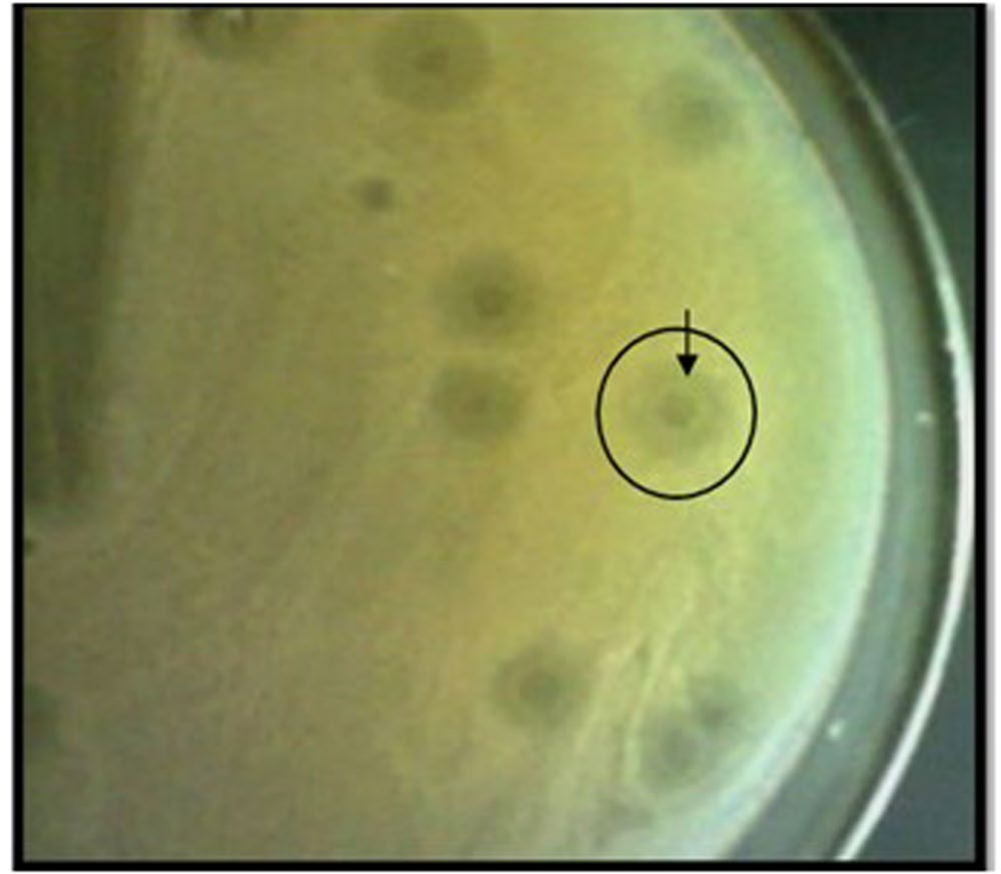
Plaque morphology. Plaques (clear surrounded by a large halo, 3 mm diameter) formed by TSK1 bacteriophage with *k*. *pneumoniae* lawn on double layer agar plate.

The bacteriophage TSK1 exhibiting potent lytic activity against *K*. *pneumoniae* was isolated from sewage water sample of TECH Housing Society Lahore. TSK1 bacteriophage formed bull’s eye shaped clear plaques (3 mm in diameter) that were surrounded by large opaque halo zone (Fig. 1). Plaque, characterized by morphology, was purified and amplified for further analysis.

### TSK1 showed highest stability at pH 7 and 37 °C temperature

Effect of different pH and temperatures on the stability of TSK1 was observed by incubating at different pH and temperatures for 1 hour followed by determination of bacteriophage titer after neutralizing the pH. Bacteriophage TSK1 showed the highest titer at pH 7 (2.7 × 10^8^ pfu/ml), while the reduction of titer was observed at pH 6, 8 and 9, whereas no stable virus was observed at pH 5 and 10 (Supplementary Fig. 1). However, the highest viral titer was noted after incubation at 37 °C (3.2 × 10^8^), than the other tested temperatures (25, 45, 55 & 60) (Supplementary Fig. 2). TSK1 bacteriophage showed narrow host range against different clinical isolates of *K*. *pneumoniae*, determined by spot test (Supplementary Table 2). One- step growth curve was performed to determine the burst size and latent period of TSK1 bacteriophage. The latent period of TSK1 was 30 minutes while the burst size was 113 pfu/infected cell (Supplementary Fig. 4). Bacterial growth reduction assay holds a tremendous relation towards phage therapy as it shows the reduction of bacterial growth through incubation time with bacteriophages. The *Klebsiella pneumoniae* infected with TSK1 bacteriophage had slower growth from the start to 14 hours of incubation. Then the growth increased gradually but remained lower than the control values (Supplementary Fig. 5).

### Sequence and phylogenetic analysis of TSK1 genome

**Figure 2:**
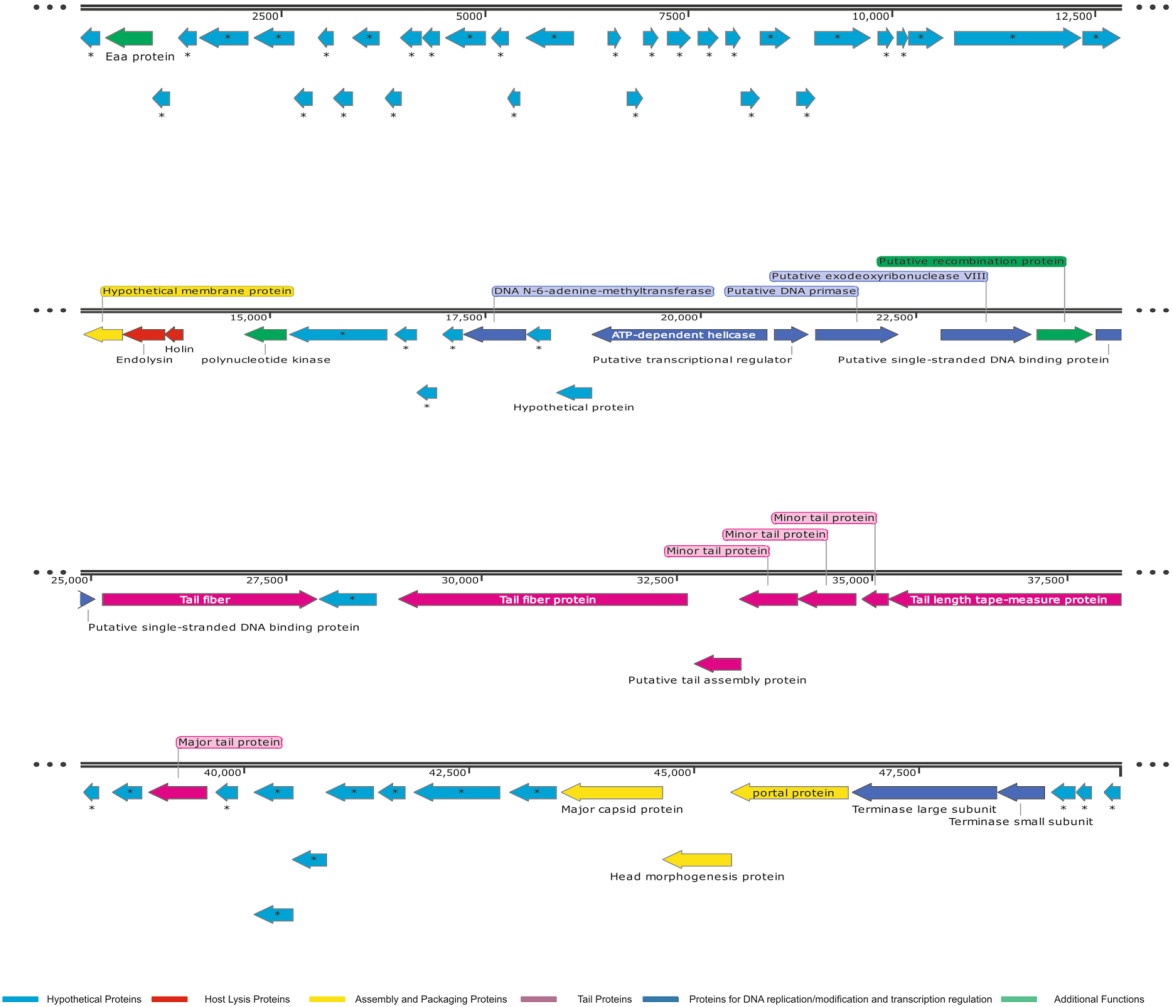
Linear genomic map of TSK1 bacteriophage obtained by Snapgene viewer. Arrow represent predicted ORFs, the direction of arrow represent direction of transcription. Different functional groups of TSK1 bacteriophage are denoted by different colors, according to their function.

DNA genome of TSK1 is 49836 bp long with GC content of 50.44%, similar to previously sequenced *K*. *pneumoniae* phages KP1513 (97% identity) and KP36 (96% identity)^16^. The genome comprising of 75 predicted open reading frames (ORFs), while no tRNA gene was predicted. Each ORF has a start codon, stop codon, ribosomal binding sites with higher Shine Dalgarno (SD) score and the minimum length of the protein-coding gene is about 120 bp (40 codons). Among 75 predicted ORFs, only 25 showed homology with known functional proteins, while remaining 50 were termed hypothetical due to the absence of any homologous gene in the genome databases. The genome annotation information of functional ORFs is presented in (Supplementary Material Table 3). The functional ORFs were further divided into five functional groups: DNA replication/modification/transcriptional regulations (DNA primase/helicase, exodeoxyribonuclease, DNA terminase, DNA N-6 methyltransferase, ssDNA binding protein, DNA adenine methyltransferase), structure and packaging (portal protein, major capsid protein, membrane protein and head morphogenesis protein), host lysis (holin, endolysin) tail structure (major and minor tail fiber protein, tail assembly and tail length tap measure proteins) and some additional proteins (recombination protein, polynucleotide kinase and Eaa like protein). These results show that the bacteriophage genome contains all the major structural and functional proteins. The orientation of genome annotation showed that most genes (53) are on the plus strand, while 22 on the reverse strand. The linear genome map with different families of proteins with known and unknown functions is presented in Fig. 2. The PhageTerm analysis showed that the genome packaging of TSK1 bacteriophage takes place through headful packaging by using the pac-site. According to the results obtained from PhageTerm, the TSK1 phage genome packaging starts at a specific packaging initiation sequences (5′ TGATCA 3′) at pac-site on concatameric precursors, like the reported P1 bacteriophage^17^. Headful packaging in TSK1 genome occurred in the forward direction. The BlastN analysis revealed that the most related genome sequences to TSK1 genome in the database were Klebsiella phage KP1513 (accession KP658157.1, 85% query coverage and 97% identity) and Klebsiella phage KP36 (accession JF501022.1, 86% query coverage and 96% identity). The results of sequence similarity suggested that TSK1 phage is a member of *Siphoviridae* family^18^.

**Figure 3:**
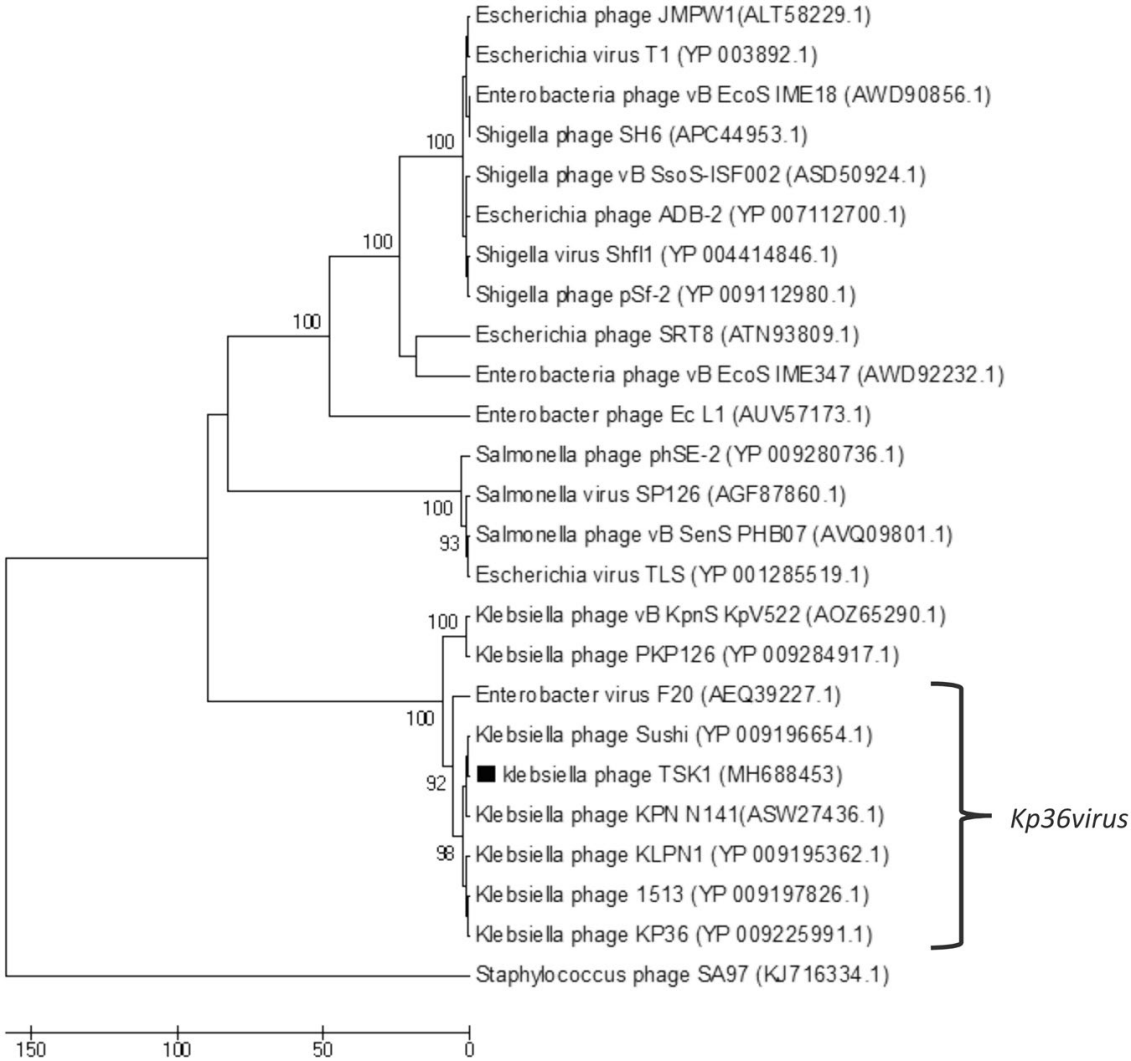
The Phylogenetic tree of TSK1 bacteriophage terminase large subunit. The tree was constructed by using the UPGMA method with 2000 bootstrap replications. The terminase large subunit of *Staphylococcus* phage SA97 was used as an out-group. The GenBank accession numbers are also provided after phage names, in parentheses. This tree showed the relationship of TSK1 terminase large subunit with other closely and distant bacteriophages.

**Figure 4:**
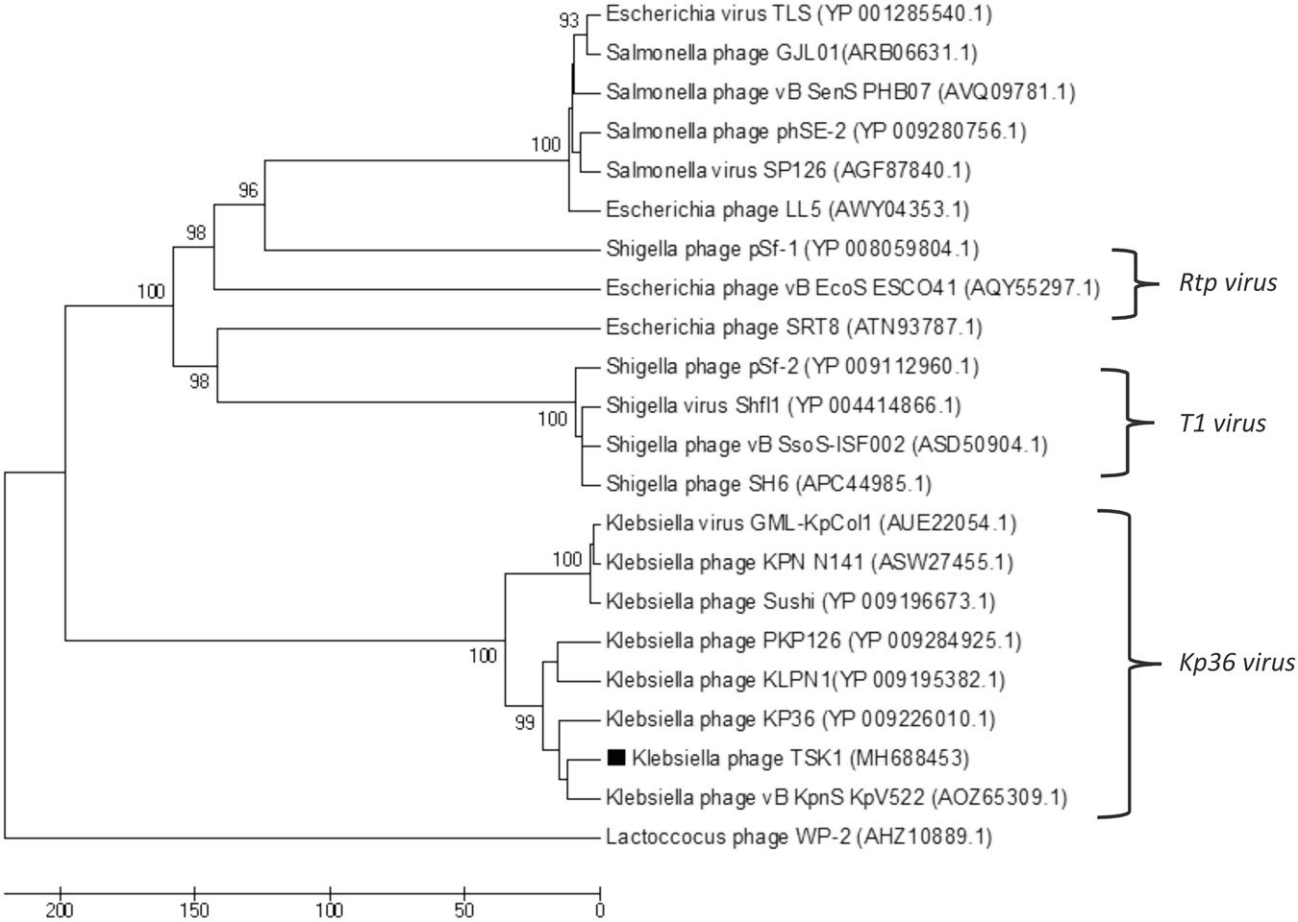
The Phylogenetic tree of TSK1 bacteriophage tail fiber protein. The tree was constructed by using the UPGMA method with 2000 bootstrap replications. The tail fiber protein of *Lactococcus garvieae* phage WP-2 was used as an out-group. The GenBank accession numbers are also provided after phage names, in parentheses. This tree showed the relationship of TSK1 tail fiber protein with other closely and distant bacteriophages.

Evolutionary relationship of TSK1 bacteriophage terminase large subunit and tail fiber protein with related phages were analyzed by using MEGA v 7.0. The amino acid sequence of TSK1 terminase and tail fiber were analyzed using BLASTp to identify putative homologs in the NCBI protein database. The phylogenetic tree showed that this phage had homology to subfamily *Tunavirinae* phages (*Klebsiella* phage 1513, *Klebsiella* phage KLPN1, *Klebsiella* phage KP36, *Klebsiella* phage PKP126 and *Klebsiella* phage Sushi). According to UPGMA dendrograms, *Klebsiella* phage TSK1 was classified as a *Kp36virus* genus in the *Tunavirinae* subfamily (Figs 3 and 4).

**Figure 5:**
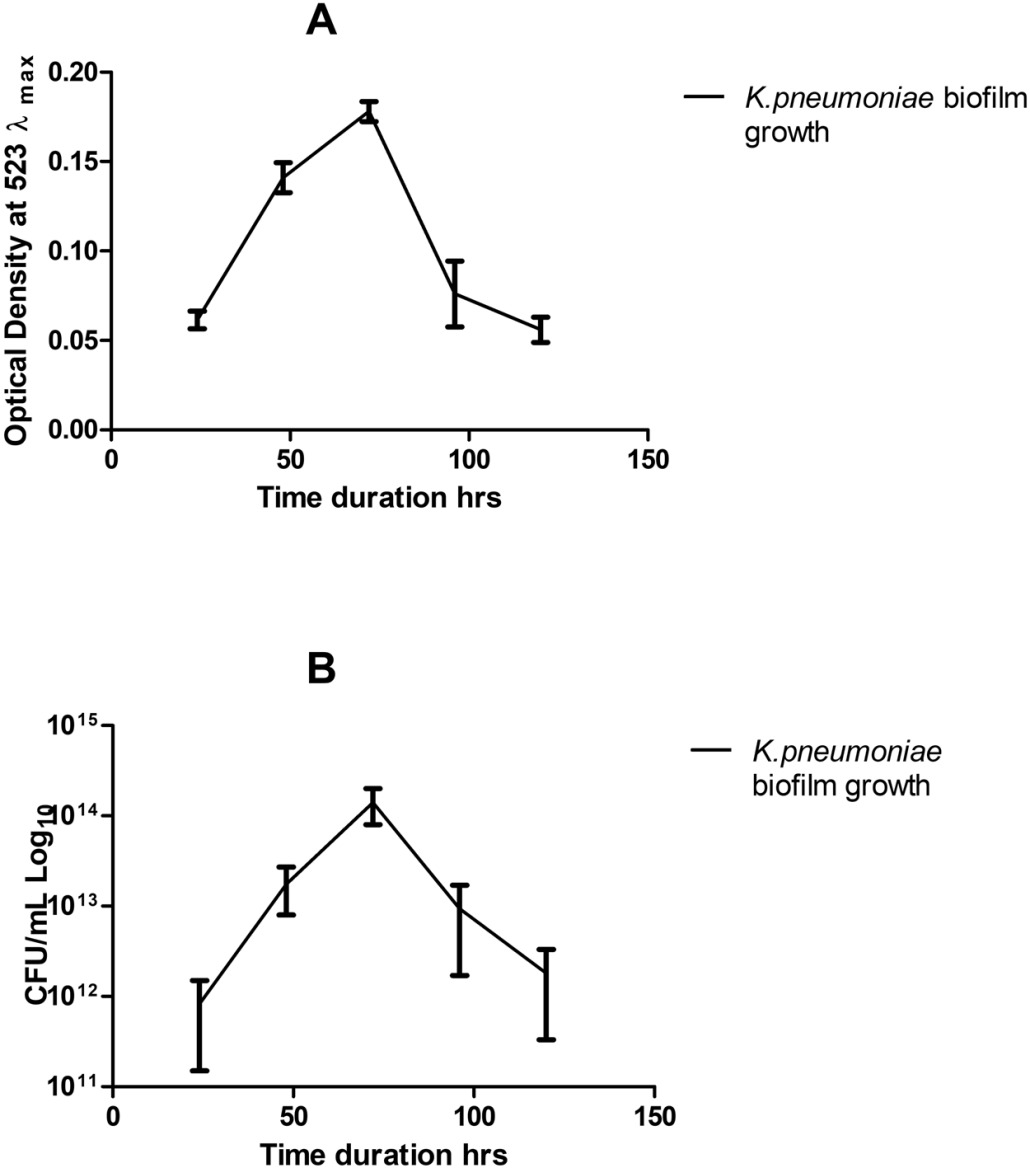
Evaluation of biofilm establishment in polystyrene microtitre plate through CV assay and Viable cell count. Different days old biofilm analyzed through CV assay (**A**) and viable cell counting (**B**).

### *K*. *pneumoniae* biofilm formation was maximum at 72 hr post- inoculation

**Figure 6:**
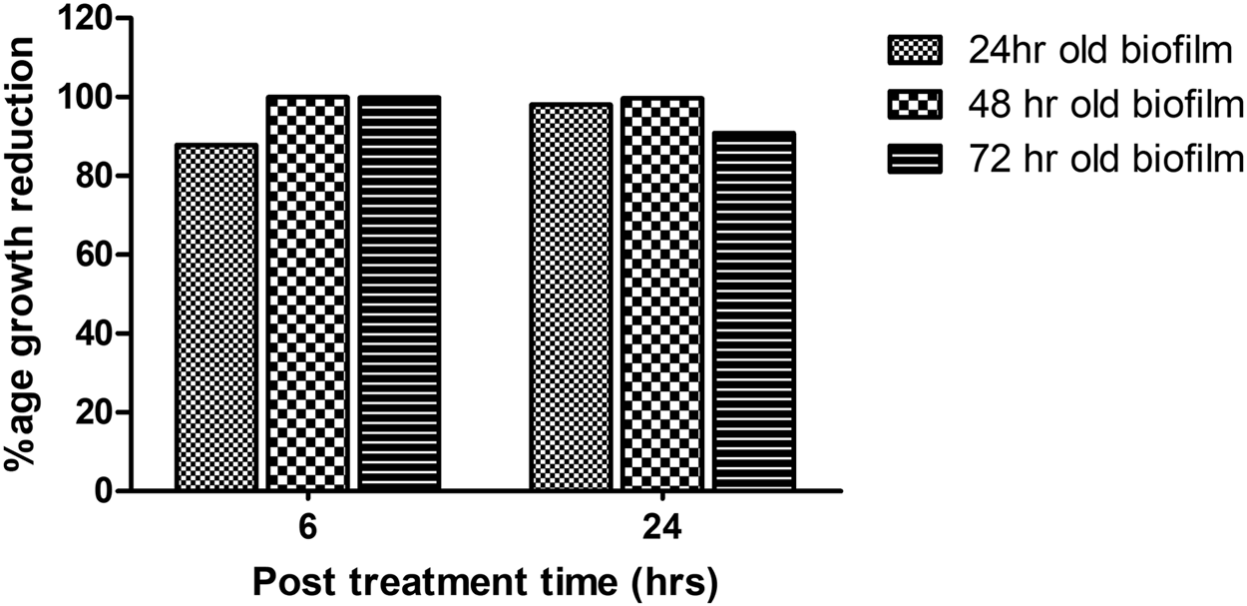
Comparison of percentage reduction in *K. pneumoniae* biofilms (1- to 3-day old) subjected to TSK1 challenge for 6 and 24 hours.

Biomass production of *K*. *pneumoniae* was analyzed by CV assay at various time points. Maximum biofilm formation was observed after 72 hr, while a decline of biomass started on further incubation (Fig. 6A). Similar observations were got in viable cell count assay. The bacterial count for young immature 24 hr old biofilm was 7.5 × 10^8^ cfu ml^−1^ followed by a maximum biomass production on the 3^rd^ day (1.4 × 10^14^ cfu ml^−1^) and a further decline resulted in a bacterial count of 1.32 × 10^12^ on the 5^th^ day of mature biofilm (Fig. 5). Based on these findings, all further experiments of biofilm eradication through TSK1 were performed on up to 72 hr old biofilms.

### Effect of exposure time on biomass reduction of K. pneumoniae biofilm by TSK1 bacteriophage

The efficient reduction of biofilm depends on the efficiency of bacteriophage invasion. The biofilm of different ages might influence the infection efficiency of bacteriophage. Significant biofilm reduction was observed upon challenging pre-formed biofilm of different ages with phage TSK1 (MOI: 0.6), at 6 and 24 hr post challenge. More than 95% biomass reduction was observed in 24 hr, 48 hr and 72 hr old biofilm treated with TSK1. There was no major difference in the biofilm removal capacity of TSK1 when applied to the biofilm of different ages (P = 0.534). The ability of the TSK1 phage to remove a bacterial load from the biofilm of different ages was 91% to 100%. The bacterial load from 48 hr old biofilm was consistently removed at 6 hr and 24 hr post inoculation, while the reduction was variable on 24 hr and 72 hr old biofilm, however still the percentage reduction was higher than 85% (Fig. 6). However, the difference between the bacterial load reductions was statistically non-significant when calculated at 6 and 24 hr post exposure (P = 0.95).

### Pre-treatment of TSK1 showed inhibition of biofilm formation

**Figure 7:**
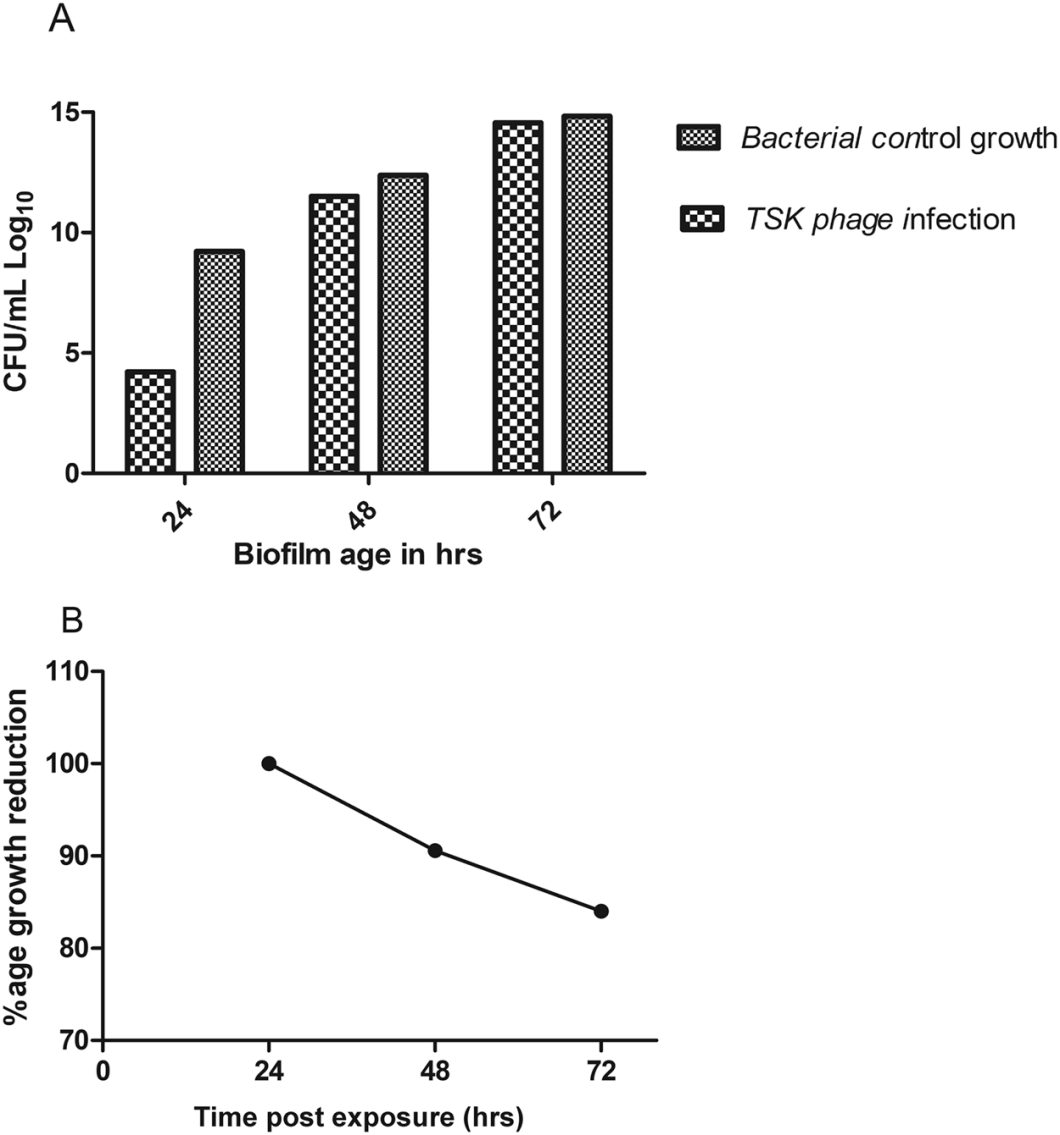
Study of Phage treatment on the pre-biofilm formation. (**A**) Bacteriophage TSK1 challenge was given at 0 times of biofilm formation and studied for 72 hr. (**B**) The percentage reduction of bacterial load compared with untreated control at different time intervals. Bacterial count was performed at 24, 48, and 72 hr post inoculation.

Pretreatment of surface with TSK1 before biofilm development showed promising results. Average percentage reduction of the bacterial load at 24, 48 and 72 hr post inoculation was 99.9%, 90% and 84% respectively (Fig. 7). Percentage reduction in the biofilm at 1–3 days post inoculation was statistically significant (P => 0.0001) at different time intervals (24 hr, 48 hr, and 72 hr).

## Discussion

Due to excessive use of antibiotics in hospitalized patients, *K*. *pneumoniae* exhibits resistance against many antimicrobial drugs. Previous studies explained that multiple drug resistant (MDR) *K*. *pneumoniae* exhibits several mechanisms such as the production of extended spectrum β-lactamases and carbapenemase^19^. The MDR strains of *K*. *pneumoniae* cause different infections in humans, which are difficult to control by using antibiotics. So, bacteriophage therapy is an effective alternative treatment option against multiple drug-resistant pathogens. In the present study, a lytic bacteriophage (TSK1) against *K*. *pneumoniae* was isolated from a sewage sample and then characterized for different physiological parameters and the whole genome. The *K*. *pneumoniae* specific bacteriophage TSK1 has the distinctive character to form opaque halo zone surrounding the clear zone of plaque. The hazy halo zones suggest the ability of TSK1 phage to produce soluble, polysaccharide-degrading enzymes^20^. Previous research showed that the formation of halo zones is an indicator for the presence of phage tail-associated exopolysaccharide (EPS) depolymerases^9^. When the bacterial growth cycle enters the stationary phase, phage replication often stops or slows down substantially but their tail spikes still can depolymerize bacterial exopolysaccharide, resulting in the appearance of an increased transparent zone known as the halo zone^9^. It was deduced from previous research that phage polysaccharide depolymerase can degrade bacterial capsules^21^. Verma and colleagues reported that bacteriophage ФNDP with no depolymerase was unable to significantly decrease *K*. *pneumoniae* biofilm mass, however, the bacteriophage KPO1K2 with depolymerase activity was able to significantly decrease biofilm mass^22^. Additionally, the halo zones could also be due to diffusion of virus-encoded non-virion associated lytic enzymes such as endolysins, which might have tried to degrade the cell wall of neighboring cells^23^.

Both temperature and pH are crucial factors that influence the bacteriophage stability. Temperature affects the whole phage replication process and also regulate phage viability, occurrence, and storage^24^. TSK1 showed the highest activity at 37 °C while a reduction in phage titer was observed at all other temperatures (Supplementary Fig. 2). The phage was completely inactivated at 65 °C temperature, similar to *Kp34virus* genus phages as described earlier^25^. It showed more activity at alkaline pH than acidic pH (Supplementary Fig. 1), however, 7.0 pH found optimal for this phage. TSK1 is not suitable for direct oral delivery of the phage for therapeutic purpose without some kind of protective layers such as alginate and chitosan^26^ since the baseline pH of 1.5 in the human stomach^27^ will deactivate the phage. Due to its stability at alkaline pH, it can be used as a therapeutic agent for *K*. *pneumoniae* mediated urinary tract infection (UTI) and infected wounds. It can be used in the impregnation of urinary catheters to inhibit bacteriological biofilm as previously suggested^28^. TSK1 phage was examined for its lytic ability against 16 strains belonging to *K*. *pneumoniae* and other related and unrelated genera. TSK1 showed activity against few tested strains of *K*. *pneumoniae*, while not showed lytic activity against tested members of other genera; depicting its narrow host range (Supplementary Table 2). These results demonstrate that TSK1 phage showed host specificity within the genus *Klebsiella*. Previously, the bacteriophages KP1513 and KP-34 against *K*. *pneumoniae* also showed narrow host range^25,29^. TSK1 can be applied in phage therapy in combinations with other bacteriophages as phage cocktails and with antibiotics for the eradication of *K*. *pneumoniae* infections. The one-step growth curve of bacteriophage reveals that the latent period for TSK1 phage is short with moderate burst size. These results clearly indicate that TSK1 possesses replication properties similar to phage B5055^28^. The application of phages in phage therapy and their feasibility can be predicted by the results of bacterial growth reduction assay. TSK1 phage reduced the bacterial growth till 14 hours, while the appearance of resistance in bacteria started at 14 hr, post-treatment (Supplementary Material Fig. 4), which indicates the development of bacteriophage resistant mutants. The exact mechanism of bacteriophage resistance is not clear, however different mechanisms like downregulation of bacteriophage receptors and CRISPR/Cas mediated digestion of bacteriophage genome, production of proteins to abort bacteriophage replication and others are proposed which need further exploration^30^.

The whole genome sequencing and genome annotation of TSK1 bacteriophage revealed that it has a 49,836 bp long genome, having 75 ORFs and no tRNA gene. The predicted homology of protein-coding sequences (CDSs) with genes from reported whole genomes showed maximum resemblance with *Siphoviridae* bacteriophages KP36 and KP1513^16^. Unsurprisingly, it did not show any evolutionary relationship with the members of the family *Podoviridae* and *Myoviridae*. The genome annotation of TSK1 bacteriophage showed that it has all basic structural and functional genes that encode host lysis proteins, DNA replication/modification and packaging proteins, tail structure proteins and some additional functional proteins. The gene products with potential antibacterial properties could be used in phage therapy in the future^31^. A large portion of the genome (67%) represents hypothetical proteins (HPs). There are four ORFs, which not showed any homology to sequences in databases while remaining forty-nine ORFs showed homologies to the HPs of *Siphoviridae* phages. More than half of a *K*. *Pneumoniae* phage JD001 genome also comprised of functionally uncharacterized hypothetical proteins^32^. Furthermore, novel HPs may also serve as markers and pharmacological targets for drug design, discovery, and screening^33^. Phylogenetic trees of terminase large subunit and tail fiber protein of TSK1 bacteriophage and other similar *K*. *pneumoniae* phages showed that the TSK1 proteins showed homology with other Klebsiella phages of *Kp36 virus* genus (Figs 3 and 4). This indicates that TSK1 is a novel virulent bacteriophage of a proposed new genus called Kp36 like virus. The bioinformatics approach of utilizing PhageTerm for identifying termini of the bacteriophage genome has identified its genome as a linear molecule with “pac” site and “headful packaging” strategy, also reported for P1and T1 type viruses^34^. The proposed packaging of TSK1 occurs in the forward direction in a headful of concatameric genomic DNA and only in the direction of concatamers^35^. Terminases are involved in the cutting of concatameric DNA at specific locations initiated in a processive series from a specific packaging sequence also described earlier in P1 and T1 phages. This packaging series initiation site is called as “pac-site”, which the terminase recognize and starts a headful packaging series. The Terminase large (TerL) protein of TSK1 phage showed homology to the TerL of phages that use a pac-headful DNA packaging mechanism^36^.

Biofilm formation and development is a significant virulence factor of numerous microbial species. Biofilm-forming bacteria were found to inhibit the effectiveness of antibiotic treatment by reducing its penetration to bacteria and exhibiting the ability to evade from host responses. In this scenario, bacteriophages can be an effective alternative as they not only replicate at the site of infection but also produce special enzymes/proteins which disrupt the biofilm structure. In the present investigation, *K*. *pneumoniae* biofilm kinetics study was carried out through viable count assay. A viable count assay provides the direct estimate of live cells. However, the method is laborious and needs a lot of medium & glassware. In the current study, the viable count assay was carried out through the micro-drop method^37^. The TSK1 bacteriophage showed degradation of different age biofilms (24, 48 and 72 hr old), and showed >95% biomass reduction during 24 hr post-treatment. The biofilm removal tendency was not different for biofilm of different ages as the difference in biomass reduction was not statistically significant (P = 0.534). Similarly, the biomass reduction capability also not came out different when tested for different incubation times (6 and 24 hr post exposure, P = 0.95). The pre-treated surfaces with bacteriophage TSK1 also showed decreased development of biofilm by *K*.*pneumoniae* for three different incubation durations (24, 48 and 72 hr) post inoculation. Amazingly the biofilm inhibition tendency was decreased with the passage of time (99.9% to 84%) and the difference in biofilm inhibition tendency at different incubation times was statistically significant (P = 0.0001). The decrease in biofilm inhibition tendency might be due to the production of some compounds after a certain time of bacterial growth, which decreased the bacteriophage entry and needs further research. It could also be due to the emergence of bacteriophage resistant cells, which also requires further exploration. A similar pattern of decrease in biofilm inhibition capacity of a bacteriophage JHP against *Pseudomonas aeruginosa* biofilm was reported earlier^38^. A *K*.*pneumoniae* bacteriophage B5055 showed enhanced biofilm removal capacity when used in combination with Co(II) as compared with it alone^1^.

## Conclusion

The emergence of multiple antibiotic-resistant *K*. *pneumoniae* strains has limited the use of antibiotics to control this pathogen. In this study, a novel lytic bacteriophage TSK1, against multidrug-resistant *K*. *pneumoniae* was isolated from sewage water. Its narrow host range makes it capable to control specific pathogen, while its stability at various pH and temperatures, burst size and latent period makes it suitable for bacteriophage therapy. The significant novelty of this study is that TSK1 bacteriophage produces depolymerase enzyme which plays a role in the disruption of *K*. *pneumoniae* capsular polysaccharide and biofilm. The TSK1 with reported physiological and genetic characters could be a new candidate for bacteriophage therapy either singly or in the form of cocktails with other bacteriophages.

## Materials and Methods

### Bacterial strain and culture conditions

An environmental isolate of *K*. *pneumoniae* ShA2 strain (accession number **KF975429**) provided by Dr. Yasir Rehman (Assistant Professor at Department of Microbiology and Molecular Genetics, University of the Punjab, Lahore) was used as a host for isolation and characterization of bacteriophage. The strain was 16 S rRNA sequenced from Macrogen, Korea. Its sequence was found 99% identical to *K*. *pneumoniae*. The selected bacterial strain was cultured in Luria Bertani (LB) medium and incubated at 37 °C. Antibiogram of *K*. *pneumoniae* ShA2 strain is available in Supplementary Table 1.

### Bacteriophage isolation and Purification

The bacteriophage was isolated from wastewater collected from TECH Housing Society Lahore by a previously reported method^39^ with slight modification. Briefly, after centrifugation of the liquid sample, 10 ml of the supernatant was mixed with 10 ml of 2X L-broth. Then 100 µl of 6 hours fresh culture of the host bacteria was added, and the flask was incubated in a shaking incubator at 37 °C for 24 hours followed by centrifugation. The supernatant was filtered using a 0.45-μm filter and spotted onto LB plates overlaid with the respective strain to detect phage plaques. An agar overlay method was used for the isolation of a pure phage preparation and to determine phage titer. A tenfold dilution series (1–10^–9^) of lysate was prepared in Luria Bertani broth, these dilutions were mixed with an exponentially grown culture of the host bacteria and incubated at 37 °C for 15 minutes. The incubated mixture was then mixed in soft agar medium and plated over the solid agar plate. Single plaque isolation, elution, and re-plating were performed repeatedly^40^. The filtrate containing bacteriophages was subjected to the bacteriophage titer determination (pfu ml^−1^) through a double layer agar method followed by recording the plaque morphology.

### Determination of Phage host range

Host range of the isolated phage was assessed on 16 clinical isolates from a medical Microbiology lab in Lahore, through standard spot test^40^. Lytic activity was determined against *K*. *pneumoniae* and other strains listed in Supplementary Material Table 2. Briefly, a 100 ul of test strain(s) was mixed with 3 ml of semi-solid medium and poured on LB agar medium. After solidification, 10ul of isolated and purified phage lysate was spotted onto the marked area against every bacterial strain. Following overnight incubation at 37 °C, plates were observed for a clear spot in the bacterial lawn.

### Assessment of Bacteriophage Thermal and pH stability

The effect of different temperatures and pH on the stability of bacteriophage was determined as reported previously^41^. The effect of temperature on bacteriophage was studied at different temperatures (28, 37, 40, 45, 55, 60 and 65 °C). A known titer of purified bacteriophage was incubated at different temperatures for one hour followed by bacteriophage titer determination. Similarly, known phage titer was incubated at different pH values (5, 6, 7, 8, 9 and 10) for one hour at 37 °C followed by titer count after neutralizing the pH to 7.0.

### Assessment of Bacteriophage Bacterial growth reduction Tendency

The bacterial growth reduction assay was determined by an already reported method^41^. Overnight bacterial culture (1 × 10^8^ cfu) was added into two L-broth flasks (50 ml). One flask was inoculated with bacteriophage (1 × 10^8^ pfu), while the other was taken as a control and incubated at 37 °C with shaking. The optical density (OD_600_) was noted for 24 hr at an interval of 2 hours.

### One-step growth curve

One-step growth curve of isolated bacteriophage against *K*. *pneumoniae* was performed in duplicates according to the previously reported method^41^. Fresh host strain (1 × 10^6^ cfu) was mixed with bacteriophage (1 × 10^8^ pfu) at an MOI of 100 in 500 μL of L-broth and incubated at 37 °C for one minute. The mixture was centrifuged at 13,000 rpm for 30 seconds to eradicate free bacteriophages and the pellet was incubated at 37 °C after re-suspension in 100 mL of fresh broth. Samples were collected for one hour with a 5-minute interval, centrifuged and the phage titer was determined. Burst size was determined from the ratio of the mean yield of phage used for bacterial infection to the mean of phage particles liberated after infection.

### Genome characterization and analysis

The genome of TSK1 bacteriophage was extracted utilizing Phage DNA Extraction Kit (Norgen Biotek, Canada, Cat No; 46850) according to manufacturer’s protocol. Supernatant with high concentration of viruses was used to isolate the genomic DNA. Whole genome sequencing of TSK1 bacteriophage was done by next-generation sequencing from the University of Minnesota, Genomic Centre (UMGC). The qualified sequence reads were assembled using CLC genomic workbench 10. The gene annotation and phylogenetic analysis of the complete genome were performed utilizing different bioinformatics tools. PHASTER, Gene Marks and Gene Glimmer were used for identification of open reading frames (ORFs) within the genome^42,43^. The confirmation of the predicted ORF was done by finding ribosomal binding sites (RBS) using the PECAAN program [https://discover.kbrinsgd.org/autoannotate/]. The predicted ORFs were annotated for specific functions using the BLASTp [https://blast.ncbi.nlm.nih.gov/Blast.cgi?PAGE = Proteins] and InterProScan programs with various protein domain databases [http://www.ebi.ac.uk/interpro/search/sequence-search]. InterProScan is a tool that allows protein sequences to be scanned against several different databases i.e HAMAP Prosite-Profiles, PANTHER, PfamA, PIRSF, ProDOM, SMART, TIGRFAM, Prosite-Patterns and structural domains i.e Gene3d, SUPERFAMILY^44^. The proteins were characterized by their molecular weight and the isoelectric point at online Sequence Manipulation Suite [http://www.bioinformatics.org/sms2/dna_mw.html] and [http://www.bioinformatics.org/sms2/protein_iep.html] respectively. Genome map was drawn with the help of Snapgene [http://www.snapgene.com/]. DNA termini and phage packaging mechanism were determined by PhageTerm through its online application incorporated in galaxy (https://galaxy.pasteur.fr/). PhageTerm software uses raw sequence reads of a phage and its genomic reference sequence to determine the termini position^45^. The complete genome sequence of the *K*. *pneumoniae* phage TSK1 was deposited in GenBank under accession no. MH688453. The amino acid sequences of two predicted ORFs (terminase large subunit (ORF 19) and tail fiber protein (ORF 38) and other bacteriophages sequences showing maximum similarity at NCBI Blast were aligned in MEGA version 7.1. The Phylogenetic tree of the aligned sequences was constructed using the UPGMA (unweighted pair group method with arithmetic mean)^46^ method with 2000 bootstrap value. The *Staphylococcus* phage SA97 terminase large subunit and *Lactococcus garvieae* phage WP-2 tail fiber were used as an out-groups^47^ for both phylogenetic trees.

### Assessment of biofilm removal capacity of TSK1 bacteriophage

Biofilm development is an important characteristic of *K*. *pneumoniae* responsible for a wide range of nosocomial infections. The activity of TSK1 to eradicate the *K*. *pneumoniae* biofilm was assessed. To determine the effectiveness of bacteriophage TSK1 treatment for eradication of *K*. *pneumoniae* biofilms, the biofilms were grown in microtiter plates. The biofilm was developed by adding LB broth (196 µl) and 4 μl of bacterial culture (10^12^ cfu ml^−1^) in the wells of a pre-sterilized microtiter plate, followed by incubation at 37 ± 1 °C for up to five days.

### Kinetics of biofilm formation

To study the kinetics of biofilm formation, viable cell count was performed according to the method described earlier^38^. In this method, after every 24 hours, planktonic bacteria were removed and a set of two control and two wells of a microtiter plate (corresponding to each day) were washed thoroughly three times with 0.85% NaCl. The adherent biofilm was incubated with sterile 0.85% NaCl for 10 min on the rocker. The suspension was vortexed for 3 min using Cyclomixer (Remi Instruments & Appliances, Germany). For the quantitative measurement of biofilm through the viable count, the homogeneous biofilm suspension was serially diluted and micro drop method^37^ was carried out on L. agar plates followed by incubation at 37 ± 1 °C for 24 hr. This procedure was repeated until the 5^th^ day of the experiment.

### Bacteriophage treatment of biofilm grown on microtiter plates

Two types of phage treatments were carried out to check the susceptibility of biofilm from bacteriophage TSK1: Post-treatment of phage infection and pre-treatment of phage infection. In Post-treatment phage infection experiments, *K*. *pneumoniae* biofilm of different ages (1–3 days old) grown in a microtiter plate was exposed to bacteriophage at 0.6 MOI. The viable count was determined after 6 and 24 hr of incubation in the treated and untreated biofilm. The reduction in log values of the bacterial count was reported compared with untreated control. In the pre-treatment experiments, LB broth containing phage TSK1 was added in the wells of the microtiter plate along with the bacterial culture at an MOI of 0.6 and incubated the plate at 37 ± 1 °C for 72 hr. A Viable count was determined after every 6 and 24 hr time interval, followed by the comparison of reduction in log values of the bacterial count with untreated control. The percentage reduction in a particular treatment was calculated by the following formula.

$${\rm{Percentage}}\,{\rm{reduction}}=({\rm{1}}-T/U)\times 100$$

Where T = bacterial load in the treated sample and U = Bacterial load in the untreated sample

### Statistical analysis

All experiments were performed in duplicates. On different days of biofilm formation, all the data from a particular treatment and time points were grouped and log reductions compared to untreated biofilm at the respective time points were calculated. The effect of different treatments on biofilm eradication was tested by GraphPad Prism 6 using an appropriate test (unpaired t-test and one way ANOVA) at a confidence interval of 95%.

## Electronic supplementary material

Supplementary Dataset 1

## Data Availability

The data is submitted with the Accession number no. MH688453, however it will be public on publication of the paper.
