# Supplementary material for: Complete genome analysis of a Siphoviridae phage TSK1 showing biofilm removal potential against Klebsiella pneumoniae
Source: Sci Rep. 2018 Dec 17;8:17904. doi: 10.1038/s41598-018-36229-y (PMC6297243; doi:10.1038/s41598-018-36229-y)
Supplement: Supplementary file 1 — Supplementary Dataset 1 [file 41598_2018_36229_MOESM1_ESM.docx]

**Complete genome Analysis of a Siphoviridae Phage TSK1 showing biofilm removal potential against *Klebsiella pneumoniae***

Rabia Tabassum, Muafia Shafique^1,2^, Komal Ameer Khawaja^1^, Iqbal Ahmed Alvi^1,3^, Yasir Rehman^1^, Cody S. Sheik^4^, Zaigham Abbas^1^, Shafiq ur Rehman^1*^

1. Department of Microbiology and Molecular Genetics, University of the Punjab, Lahore, Pakistan
2. Food and Biotechnology Research Centre, PCSIR Laboratories Complex, Lahore, Pakistan
3. Department of Microbiology, Hazara University Mansehra, KPK Pakistan
4. Swenson College of Science and Engineering, University of Minnesota Duluth, US

**Correspondence:** Dr. Shafiq ur Rehman, Assistant Professor, Department of Microbiology and Molecular Genetics, University of the Punjab, Lahore, Pakistan: email: shafiq.mmg@pu.edu.pk

**SUPPLEMENTARY MATERIAL**

**Figure 1.**

**
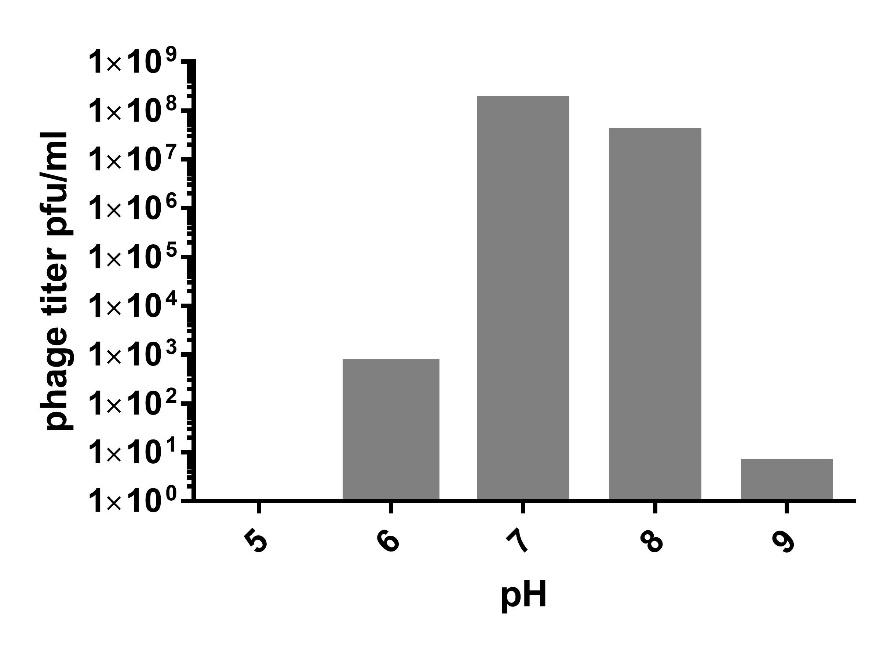
**

**Figure 2.**

**
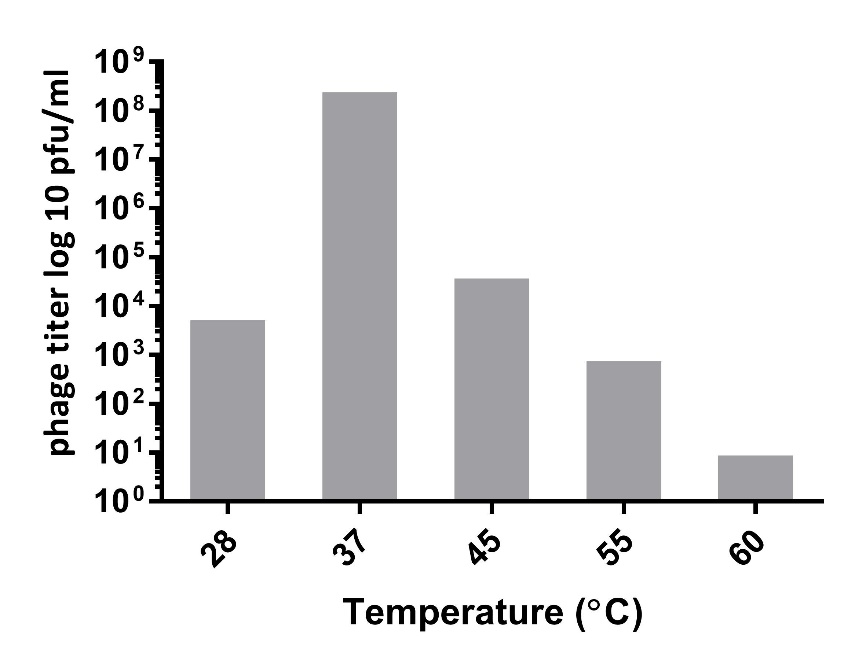
**

**Figure 3.**


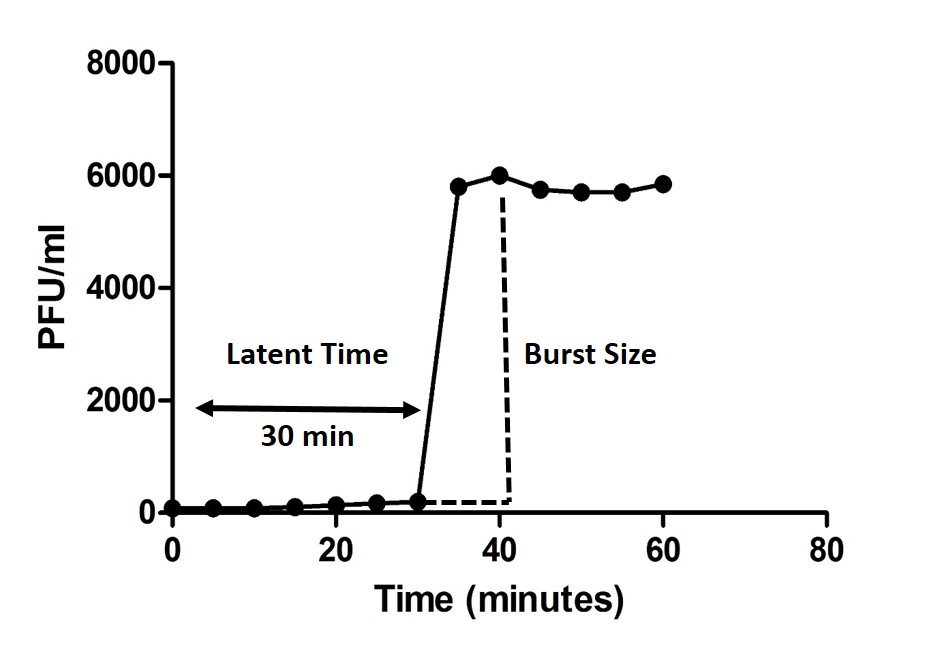


**Figure 4.**


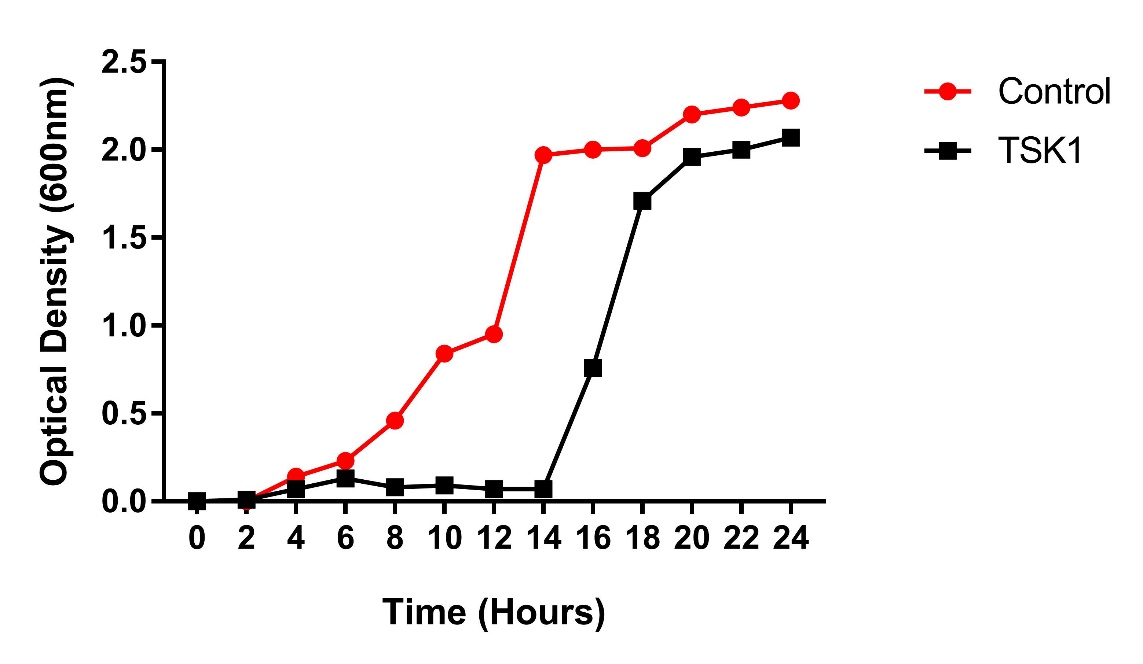


**Table and Figure legends**

**Table.1.** Antibiogram of *Klebsiella pneumoniae 1* (ShA- 2)

**Table.2.** Host range determination for TSK1 bacteriophage

**Table.3.** BLASTp results for protein sequence analysis of *Klebsiella pneumoniae* bacteriophage (TSK1)

**Figure 1**. Effect of pH on stability of TSK1 bacteriophage treated at different pH for an hour

**Figure 2.** Effect of temperature on stability of TSK1 bacteriophage treated at different temperatures for an hour

**Figure 3.** One Step Growth Curve of TSK1 Bacteriophage

**Figure 4.** *K. pneumoniae* growth reduction by TSK1 bacteriophage

**Table.1.**

| **Antibiotics** | **Antimicrobial Susceptibility/Resistivity of ShA-2** |
| --- | --- |
| Chloramphenicol (30 ug/ml) | R |
| Ampicillin (10 ug/ml) | R |
| Oxytetracycline (30 ug/ml) | S |
| Streptomycin (10 ug/ml) | S |
| Erythromycin (15 ug/ml) | R |
| Kanamycin (30 ug/ml) | S |

**Table.2.**

| **Bacterial strain** | **Spot test** | **Source** |
| --- | --- | --- |
| *Klebsiella pneumoniae 1* (ShA- 2) | **+** | wounds |
| *Klebsiella pneumoniae 2* | **+** | wounds |
| *Klebsiella pneumoniae 3* | **-** | UTI |
| *Klebsiella pneumoniae 4* | **-** | UTI |
| *Klebsiella pneumoniae 5* | **+** | UTI |
| *Klebsiella pneumoniae 6* | **-** | UTI |
| *Klebsiella pneumoniae 7* | **-** | wounds |
| *Klebsiella pneumoniae 8* | **+** | wounds |
| *Escherichia coli 1* | **-** | UTI |
| *Escherichia coli 2* | **-** | UTI |
| *Escherichia coli 3* | **-** | Wounds |
| *Pseudomonas aeruginosa* | **-** | Burn wounds |
| *Enterobacter cloacae 1* | **-** | UTI |
| *Enterobacter cloacae 2* | **-** | RTI |
| *Acinetobacter baumannii* | **-** | RTI |
| *Staphylococcus aureus* | **-** | wounds |

- was unable to produce lytic zone

+ produced clear lytic zon**e**

**Table 3.**

| **S.No** | **ORF & CDS Position** | **Predicted function** | **Length (number of amino acids)^a^** | **% identity** | **BLASTp best match** | **Accession no** | **Isoelectric point**  **(pI)** | **Molecular weight**  **(KDa)** |
| --- | --- | --- | --- | --- | --- | --- | --- | --- |
| 1 | TSK1_00011  (3831..4022) | Membrane protein | 63 | 63/63  (100%) | Hypothetical Membrane protein [Klebsiella phage Sushi] | YP_009196678.1 | 8.03 | 7.11 |
| 2 | TSK1_00013  (4343..4918) | Membrane protein | 191 | 188/191  (98%) | EaA protein [Klebsiella phage 1513] | [YP_009197832.1](https://www.ncbi.nlm.nih.gov/protein/YP_009197832?report=genbank&log$=protalign&blast_rank=1&RID=BPBZXZ48013) | 6.04 | 21.70 |
| 3 | TSK1_00018  (6104..6628) | Terminase small subunit | 174 | 172/174  (99%) | Terminase small subunit [Klebsiella phage Sushi] | [YP_009196653.1](https://www.ncbi.nlm.nih.gov/protein/YP_009196653.1?report=genbank&log$=protalign&blast_rank=2&RID=NM2W25FS015) | 5.37 | 19.68 |
| 4 | TSK1_00019  (6638..8239) | Terminase large subunit | 533 | 532/533  (99%) | Terminase large subunit [Klebsiella phage Sushi] | YP_009196654.1 | 6.69 | 61.44 |
| 5 | TSK1_00020  (8285..9595) | Portal protein | 436 | 431/436  (99%) | Portal protein [Klebsiella phage KP36] | YP_009225992.1 | 4.62 | 48.07 |
| 6 | TSK1_00021  (9585..10352) | Head morphogenesis protein | 255 | 247/255  (97%) | Head morphogenesis protein [Klebsiella phage KP36] | YP_009225993.1 | 6.56 | 29.04 |
| 7 | TSK1_00022  (10349..11482) | Major capsid protein | 377 | 376/377  (99%) | Major capsid protein [Klebsiella phage KP36] | YP_009225994.1 | 4.90 | 41.27 |
| 8 | TSK1_00030  (15416..16072) | Major tail protein | 218 | 214/218  9(98%) | Major tail protein [Klebsiella phage 1513] | YP_009197815.1 | 4.90 | 24.06 |
| 9 | TSK1_00033  (16826..19792) | Tail length tape-measure protein | 988 | 973/988  (98%) | Tail length tape-measure protein [Klebsiella phage KP36] | YP_009226005.1 | 9.57 | 107.09 |
| 10 | TSK1_00034  (19795..20139) | Minor tail protein | 114 | 109/114  (96%) | Minor tail protein [Klebsiella phage Sushi] | YP_009196669.1 | 7.25 | 12.33 |
| 11 | TSK1_00035  (20209..20961) | Minor tail protein | 250 | 247/250  (99%) | Minor tail protein [Klebsiella phage KP36] | YP_009226007.1 | 7.74 | 27.51 |
| 12 | TSK1_00036  (20963..21700) | Minor tail protein | 245 | 242/245  (99%) | Minor tail protein [Klebsiella phage 1513] | [YP_009197809.1](https://www.ncbi.nlm.nih.gov/protein/YP_009197809?report=genbank&log$=protalign&blast_rank=2&RID=BPSVX0V1016) | 6.32 | 28.47 |
| 13 | TSK1_00037  (21675..22277) | Putative tail assemblage protein | 200 | 189/200  (95%) | Putative tail assembly protein [Klebsiella phage KP36] | YP_009226009.1 | 10.21 | 20.83 |
| 14 | TSK1_00038  (22365..26084) | Tail fiber protein | 1233 | 1131/1227  (92%) | Tail fiber protein [Klebsiella phage KP36] | YP_009226010.1 | 4.65 | 137.50 |
| 15 | TSK1_00041  (30063..30527) | Putative single stranded DNA binding protein | 154 | 151/154  (98%) | Putative single-stranded DNA binding protein [Klebsiella phage KP36] | YP_009226012.1 | 5.70 | 17.62 |
| 16 | TSK1_00042  (30564..31220) | Putative recombination protein | 218 | 216/218  (99%) | Putative Recombination protein [Klebsiella phage KLPN1] | [YP_009195385.1](https://www.ncbi.nlm.nih.gov/protein/YP_009195385?report=genbank&log$=protalign&blast_rank=4&RID=BR7ZV6JR014) | 6.81 | 24.34 |
| 17 | TSK1_00043  (31280..32326) | Putative exodeoxyribonuclease VIII | 348 | 343/348  (99%) | Putative exodeoxyribonuclease VIII [Klebsiella phage KP36] | YP_009226014.1 | 5.29 | 39.23 |
| 18 | TSK1_00044  (32822..33781) | Putative DNA primase | 319 | 312/319  (98%) | Putative DNA primase [Klebsiella phage KP36] | YP_009226015.1 | 6.11 | 36.08 |
| 19 | TSK1_00045  (33857..34258) | Putative transcriptional regulator | 133 | 133/133  (100%) | Putative transcriptional regulator [Klebsiella phage 1513] | YP_009197873.1 | 11.33 | 15.21 |
| 20 | TSK1_00046  (34350..36383) | ATP-dependent helicase | 677 | 670/677  (99%) | ATP-dependent Helicase [Klebsiella phage KP36] | [YP_009226017.1](https://www.ncbi.nlm.nih.gov/protein/YP_009226017?report=genbank&log$=protalign&blast_rank=3&RID=BR8FKXR4014) | 7.57 | 77.17 |
| 21 | TSK1_00049  (37145..37876) | DNA N-6 adenine-methyltransferase | 243 | 237/243  (98%) | DNA adenine methyltransferase [Klebsiella phage KP36] | YP_009226020.1 | 6.30 | 27.79 |
| 22 | TSK1_00054  (39930..40418) | Polynucleotide kinase/Phosphatase | 162 | 161/162  (99%) | Polynucleotide kinase/phosphatase [Klebsiella phage KP36] | YP_009226025.1 | 6.15 | 18.35 |
| 23 | TSK1_00056  (41123..41338) | Phage holin | 70 | 70/70  (100%) | Holin [Klebsiella phage 1513] | YP_009197862.1 | 11.01 | 7.43 |
| 24 | TSK1_00057  (41340..41822) | Endolysin | 160 | 158/160  (98%) | Endolysin [Klebsiella phage 25KP36] | YP_009226028.1 | 9.95 | 17.97 |
| 25 | TSK1_00058  (41819..42244) | Membrane protein | 140 | 140/140  (100%) | u-spanin [Klebsiella phage Sushi] | [YP_009196694.1](https://www.ncbi.nlm.nih.gov/protein/YP_009196694?report=genbank&log$=protalign&blast_rank=1&RID=BPZ13GXT013) | 9.84 | 14.74 |

**^a^ no of amino acids**
